# Connecting at Local Level: Exploring Opportunities for Future Design of Technology to Support Social Connections in Age-friendly Communities

**DOI:** 10.3390/ijerph17155544

**Published:** 2020-07-31

**Authors:** Jennifer Liddle, Nicole Pitcher, Kyle Montague, Barbara Hanratty, Holly Standing, Thomas Scharf

**Affiliations:** 1 Population Health Sciences Institute, Campus for Ageing and Vitality, Newcastle University, Newcastle upon Tyne NE4 5PL, UK; barbara.hanratty@newcastle.ac.uk (B.H.); thomas.scharf@newcastle.ac.uk (T.S.); 2 Open Lab, Newcastle Helix, Newcastle University, Newcastle upon Tyne NE4 5TG, UK; kyle.montague@newcastle.ac.uk; 3 Assistance Publique – Hôpitaux de Paris, 75004 Paris, France; nicole.valtorta@gmail.com; 4 Department of Nursing, Midwifery and Health, Coach Lane Campus East, Northumbria University, Newcastle upon Tyne NE7 7XA, UK; holly.standing@northumbria.ac.uk

**Keywords:** connectedness, social relationships, later life, ageing, older people, age-friendliness, community, digital technology, loneliness, isolation

## Abstract

Social connectedness in later life is an important dimension of an age-friendly community, with associated implications for individual health and wellbeing. In contrast with prior efforts focusing on connections at a distance or online communities where the digital technology is the interface, we explore the design opportunities and role of technology for connectedness within a geographically local community context. We present findings from interviews with 22 older adults and a linked ideation workshop. Our analysis identified shared concerns and negative perceptions around local relationships, connections and characteristics of the geographical area. However, local connectedness through technology was largely absent from day-to-day life and even perceived as contributing to disconnection. By uncovering how older adults use and perceive technology in their social lives and combining these findings with their ideas for improving local connections, we highlight the need for thoughtful consideration of the role of technology in optimising social connections within communities. Our research highlights a need for design work to understand the specifics of the local context and reduce emphasis on technology as the interface between people. We introduce an amended definition—‘underpinned by a commitment to respect and social inclusion, an age-friendly community is engaged in a strategic and ongoing process to facilitate active ageing by optimising the community’s physical, social and digital environments and its supporting infrastructure’—to conceptualise our approach. We conclude by suggesting areas for future work in developing digitally connected age-friendly communities.

## 1. Introduction

Social connectedness in later life is important for health and wellbeing. Consequently, making it easy for people to develop and maintain social relationships is a fundamental ambition of ‘age-friendly’ communities. This local, place-based, policy approach recognises that physical and social environments are key determinants of whether people remain independent, autonomous and healthy in later life.

Human–computer interaction (HCI) researchers are directing increasing attention towards the role of technology in shaping and supporting social relationships in later life. Much of this work focuses on online communities or connecting across geographical or generational distances, where digital technology is the interface or infrastructure for connection. In addition, approaches commonly place emphasis on addressing technological inexperience, or on physical or cognitive impairment and decline.

In this paper, we are interested in considering technology and connectedness in later life within a specific local context, and exploring how innovation in social connection can be age-friendly and embedded within such physical community settings. We consider older adults as a heterogeneous group, rather than a group marked by singular identities of health, cognitive status, or technological proficiency. Nevertheless, our place-based approach aims to identify common values and experiences shared by people living in the same geographical area. Life events such as retirement, along with experiences of building and maintaining social connections over the life course, will also have implications for how and why older adults wish to develop and sustain proximate relationships in particular ways.

We suggest that considering these topics enables a deeper understanding of how to design for a digitally connected age-friendly neighbourhood, where both the design process and its outputs are age-friendly. Our paper presents findings from a study comprising two phases: qualitative interviews with 22 older adults; and a linked workshop ideation process to engage interviewees in beginning to consider how connections within their local area might be enhanced over time. The contributions of our paper centre around a context-specific and bottom-up approach to designing for increased local connectedness in later life. The importance of this topic has since been emphasised by the COVID-19 pandemic, heightening awareness of the need to consider ways to maintain and create social connectedness, particularly at a local level.

Our aim is not to design a technological output. Instead, we see our approach as prioritising a crucial, and often neglected, stage in technology design, which provides important insights that would be required for any future stage of a design process that aimed to design or create an actual technology. Themes that emerged from our interviews suggest that participants viewed technology as acceptable when it filled a ‘gap’ and did not have too many negative impacts on everyday life. Our starting point for the linked workshop was to consider some of these ‘gaps’ in local connectedness that interview participants had described. The workshop activities were used to facilitate participants in thinking creatively about addressing specific local challenges, or ‘gaps’ in connectedness. In drawing together participants’ ideas about spaces, processes and mechanisms that might address these local challenges, we conclude the paper with implications that offer scope for further exploration and consideration in terms of how technology might support the operationalisation of local people’s ideas for improving face-to-face connections in age-friendly community settings.

### Related Work

Growing interest in what makes places ‘good’ to grow old in has led to an increasing focus on the ‘age-friendliness’ of different types of environments [1]. Despite variation in emphasis between models of age-friendly environments, most approaches promote consideration of how policies, services and structures can integrate physical and social environments, supporting social engagement and connection [2]. Our work adopts the following conceptual definition, with its emphasis on age-friendliness as commitment to a process rather than a standard to be reached:

‘Underpinned by a commitment to respect and social inclusion, an age-friendly community is engaged in a strategic and ongoing process to facilitate active ageing by optimising the community’s physical and social environments and its supporting infrastructure’

[3].

The adopted definition of age-friendliness shapes our research design and methods, with its emphasis on community engagement and the participation of older people in processes to optimise the environment to support social connections. We also draw on concepts from environmental gerontology, such as ‘ageing in place’ to understand the importance of the local area in older people’s lives. An overarching premise of an age-friendly community is that it is ‘friendly for all ages and not just “elder-friendly”’ [1]. Even so, the argument that older people are ‘able to remain more independent by, and benefit from, ageing in environments to which they are accustomed’ [4] makes it all the more important to consider how environments can support people ‘ageing in place’ to optimise their social connectedness within their local area. This has become even more apparent during the 2020 COVID-19 pandemic, which has exposed the need for digital connection as an alternative to face-to-face interactions. Similarly, finding new ways to connect, even with people in proximate locations, has become a greater priority.

There has also been a strong emphasis on tackling the counterparts of social connectedness—loneliness and isolation. Warnings of the ‘loneliness epidemic’ and its associated public health implications are prevalent in media discourse [5,6,7], and the UK government appointed the world’s first minister for loneliness in 2018 [8]. Accordingly, responses to the drive for increased social connection have often focused on mitigating unpleasant experiences, risks and deficits at an individual level [9]. Efforts along these lines reflect and uphold persistent ageist stereotypes that fail to acknowledge the roles that older people (can) play in communities, or their potential to contribute innovative ideas or create a voice for themselves [10,11,12]. Indeed, technology is often presented as the ideal way of solving these ‘problems’ faced by older adults [13]. Ten Bruggencate et al. draw our attention to the predominant focus on loneliness and/or isolation in studies about social technology, ageing and relationships [14]. In contrast, a growing body of work on social connectedness in later life challenges the image of older people as lonely and isolated. Population ageing is leading to increasing numbers of older people, thereby increasing the number of older people in society who experience loneliness. However, loneliness affects only the minority of older people, including the oldest old [15,16,17]. The likelihood of reporting feeling lonely decreases with age, with younger adults (16–24 years) reporting loneliness more often than those in older age groups [18]. While older adults may have smaller social networks, they are often more involved in the community than younger adults—socialising with neighbours, participating in religious organisations and volunteering [19]. However, even if social reciprocity and meaningful interactions are desired and enacted by older people, infrastructural barriers can, and do, impede the quantity and quality of such connectedness [20].

Technology offers the potential for scalable and cost-effective interventions to address barriers to connectedness. The design, or adoption, of digital technology to support social relationships in later life often results in technology being the core interface for connection between people, rather than a route to facilitating face-to-face connections by overcoming barriers. For example, online communities are promoted as presenting opportunities for older people to meet and interact with peers [21,22,23,24]. In this interfacing role, technology is a bridge across distances. Lindley et al. comment that much HCI research related to relationships focuses on ways to maintain feelings of connectedness or express intimacy at a distance [25]. Distances being bridged may be geographical, for individuals living in remote areas or wanting to connect with people with whom they share interests, friendship or familial bonds. Distances may also be generational, where, despite intentions to the contrary, technology replicates asymmetrical family interactions [23,26,27].

Growing proportions of older people are now using digital technologies. In the UK, 83% of adults aged 65–74, and 47% of adults aged 75 and over use the internet [28]. Thus, the majority rather than a minority of older people are technologically connected, suggesting a need to understand more about how this diverse population uses, and feels about, technology for connecting with others. The few studies that have explored older people’s attitudes towards, and perceptions about, communication and connection suggest that rich interactions are valued above lightweight connections offered by newer technologies [14,25,29]. Again, this work primarily considers the capacity of digital technology to bridge geographical or generational distances, where more traditional technologies such as telephone and email are often preferred. Thoughtful and meaningful interactions are the goal, and technology provides the interface. Research methods centre around questions about how older adults use, or would choose to use, technology in their social relationships. For instance, Sayago et al. report on research with 700 older people (across six studies) that examined situated technology use and the reasons why participants did, or did not, incorporate particular forms into their everyday lives [23]. In this way, technological interfaces are often in-built as fundamental foundations for designing for connection, diminishing considerations of technology in non-interfacing roles.

Research that has explored ways to improve geographically proximate connections has also tended to concentrate on a prominent role for technology, often studying online community networks. These include bespoke online communities for older adults, or those formed on more widely used social networking platforms. Righi et al. focussed on how older people’s use of social networking sites could be used to promote their involvement in both online and offline local communities [30]. While participants used, for example, Facebook to find out information about the local area, most did not post or share information or send messages to others. Instead, these interactions took the form of face-to-face conversations. On this basis, the authors conclude that proximity and face-to-face contacts should be kept in mind when designing online community networks. We would extend this argument further, to suggest reversing the design process. Such a process would design for proximity and face-to-face contact in offline communities, with technology kept in mind in a background, less visible, role.

The research described above concentrates on technology as the interface for connection between people. While the potential of technology to foster involvement in local communities has been explored, less attention has been paid to understanding and drawing on context-specific factors to develop approaches to promote connection in local areas *with*, rather than *for*, older people. This would be a fundamental approach for any community engaged in the ongoing process becoming (more) age-friendly. An effective strategy in one community will not necessarily translate to a community with different geographical, social or structural features. Likewise, the attitudes of older people towards technology will vary individually and across communities and countries. In their ‘manifesto for change’ in age-friendly cities and communities, Buffel et al. emphasise the necessity of ensuring the empowerment and recognition of older residents in order to achieve age-friendliness [31]. For these reasons, we adopted a bottom-up, place-based approach that can be responsive to local needs, preferences and resources. We recognise community as an inclusive concept, with the participation and empowerment of members (particularly older people) being fundamental to its creation and functioning [3,32,33].

The following sections present the methods and findings of our study. Our research design (in-depth interviews followed by an ideation workshop) draws on key concepts, theories, gaps and definitions in the literature outlined above. It is a bottom-up place-based approach that focuses on local needs, preferences and resources. It prioritises the participation of older people in exploring context-specific routes to local connection that present opportunities for future design of technology. We see our participants as crucial to developing ideas to increase or improve connection. As residents within the local area, they have a wealth of knowledge and experience and are best placed to identify resources, ideas and options that can lead to context-specific routes to connection.

Our overall aim within this study is to begin exploring context-specific routes to local connection that do not start the design process with attempts to design technological interfaces. Discovering issues or opportunities for increased connection at a community level is the first step in this process. These opportunities and ‘gaps’ also need to be considered alongside insights into the current practices and perceptions of older people regarding technology in their social lives. Once opportunities for increasing connection have been identified, ways to address these can then be explored by older people with local expertise and knowledge. Therefore, in practice, the workshop methods were designed after analysis of our interview data so that we could draw on the interview findings as the starting point for workshop activities and discussions. However, for structural clarity, the methods for both the interviews and workshop are presented first in this paper, followed by the findings from our analyses.

## 2. Materials and Methods: Phase One Interviews

The first phase of our study aimed to explore opportunities for designing to improve proximate social connections for older people living within a geographically identified ‘community’. We also wanted to know more about how and why research participants were using technology, or not, in their social lives. Qualitative interviews were an appropriate method for exploring these two topics, with their potential to elicit personal accounts that help people to ‘make explicit things that have hitherto been implicit—to articulate their tacit perceptions, feelings and understandings’ about their social lives and technology [34].

### 2.1. Participants and Context

The study setting was an electoral ward (district) within a city in the North of England, UK, chosen for its proximity to the research team’s institutional location. Just over 10% of the around 13,600 people living in this geographical area are aged 60 or over (compared to 23% overall in England and Wales). It is also one of the most ethnically diverse and socially deprived wards in the region [35].

Following institutional ethical approval (Ref. 13284), we recruited 22 older adults (15 women, 7 men) to take part in audio-recorded interviews. Sixteen interviews were with individual participants and three interviews were with couples living in the same household who chose to be interviewed together. Our only inclusion criterion was that participants were aged 60 or over. However, we also sought to achieve a diverse sample in terms of age, gender, ethnicity, social connectedness and living arrangements. Table 1 summarises participant characteristics. Participants were aged between 60 and 84 and had been living in the area for between seven months and 84 years. One participant was Asian and the remaining 21 participants were White. Eight participants were living alone, and the others lived with at least one other person (a spouse/partner ± extended family). With the exception of one participant who was working part-time, all participants were retired. Recruitment was via face-to-face conversations at community events and locations (such as a weekly café held in a local church) and contact details shared by community groups and organisations based in the area. We made substantial efforts to achieve a sample with greater ethnic diversity, including seeking assistance from individuals running local organisations and groups for people from non-White backgrounds, and posters in local culturally diverse food and clothing shops. We also made provisions for language translation in interviews. However, in the time available, we were unable to identify additional people from different ethnic groups who were willing to take part in an interview. Longer-term development of relationships within the community would likely be needed to increase interest and trust, which was not possible in a study of this scale.

### 2.2. Procedure and Analysis

All potential participants were given an information sheet about the study and a copy of the consent form to read. Interviews were arranged at times to suit participants, and they were offered a choice of location. One participant chose to meet for their interview in a community building and all other interviews were conducted in people’s own homes. After completing the consent form and giving an opportunity for the interviewee to ask any questions, we audio-recorded the interview with the participant’s agreement. Interviews were conducted by JL, HS or NP. We initiated the interviews with a narrative approach, asking individuals to tell the story of their social lives since they had been living in the area. This facilitated the exploration of each individual’s own concerns, meanings and priorities related to their social lives, rather than these being imposed by predetermined questions [36]. The same question was asked at the beginning of each interview: ‘Can you please tell me the story of your social life while you’ve been living in [this area]; your relationships with family, friends, neighbours and other people?’. Participants were asked to talk about any events and experiences that were important for them, and invited to take as long as long as they needed to tell their story. This narrative section of the interview was followed by supplementary probing questions to explore areas of particular interest, including the role of technology in their social lives. These questions were not pre-defined in order that interviewers were free to explore anything that they felt was of interest and relevant to the overall aims of the study, maintaining a natural and spontaneous flow within the interview. Brief reflective field notes were made by interviewers after each interview.

Electronic data files were stored in password-protected folders in the University filestore. Interview recordings were transcribed and names were anonymised. We then completed initial inductive coding [37] of the data to explore (a) opportunities to improve connections at a local level, i.e., factors that had the potential to impact negatively on people’s geographically proximate social relationships in terms of quality, quantity or satisfaction; and (b) participants’ engagement with technology in relation to their social lives generally. Codes were organised under themes, following the process outlined by Braun and Clarke [37]. For example, codes such as ‘places people used to socialise no longer exist’, ‘many buildings are not accessible’, and ‘there are few facilities’ were grouped together under the theme ‘few local places to socialise’. Coding and theme development were completed independently by two researchers (J.L., N.P.) and then discussed and refined with all members of the research team.

While all names used in this paper are pseudonyms, participants in photographs gave consent for their images to be included in research outputs.

## 3. Materials and Methods: Phase Two Ideation Workshop

The second phase of the study comprised an ideation workshop. We drew on the following conclusions from our interview analysis when designing the workshop:

- there were concerns and perceptions about local community connections and characteristics that offered opportunities for design;
- our participants predominantly used technology to connect with family, or friends at a distance; existing local technological connections in their social lives were less obvious;
- many participants were actively using a variety of technologies, but their willingness to do so depended on perceptions of unmet needs and balancing the negative aspects (additional work, potential contribution to face-to-face disconnection) in their everyday lives.

We designed the workshop to explore and generate ideas to improve and optimise social connections in the local area, focusing on four of the opportunities we identified in our interview analysis. Based on the in-depth understanding about participants’ use and perceptions of technology that we gained from the interviews, we designed ‘playful’ workshop activities that deliberately did not ask participants explicitly to consider how technology could address issues in local social connections. Instead, we wanted to begin by eliciting participants’ thoughts about the best ways to tackle these issues before considering any technological needs that arose from these suggestions. This approach avoids the tendency of previous research to foreground technology at the start of the design process. By deliberately *not* seeking to design a technology or technological interface in this study, we could instead reflect on the potential needs or roles for technology once we knew what type of interventions our participants had suggested. Our approach also fitted well with our desire to draw on participants’ knowledge, experience and capacity for creative thinking, and was in keeping with our aim of developing approaches to promote connection with, not for, older people, prioritising their participation in a bottom-up design process.

### 3.1. Participants and Context

All interview participants were sent a postal invitation to the workshop. Eleven individuals initially confirmed their availability and nine attended on the day (6 women, 3 men). These individuals were aged between 68 and 84 and had been living in the area for between 30 and 69 years. The workshop was held in a church hall in the local area and refreshments were provided.

### 3.2. Procedure and Analysis

Participants were asked to read and complete the consent form on arrival. Consent to being photographed was optional.

The workshop was structured around four opportunities to improve local social connections that we identified as themes through our interview analysis. Each theme represented shared concerns and negative perceptions about local relationships, connections and characteristics of the area that participants had talked about. The four themes were ‘few local places to socialise’, ‘not knowing neighbours well’, ‘absence of a shared community feeling’, and ‘activities on offer not always conducive to socialising or making new friends’. These themes were chosen to take forward in the workshop based on their content being both appealing and generic enough for all participants to engage with, regardless of their individual circumstances and experiences.

In line with age-friendly models, our aim was for a bottom-up approach in which workshop attendees’ participation and contributions were fundamental to the resulting design ideas [38]. Confronting ageist stereotypes, we also wanted to capitalise on participants’ creative abilities and ingenuity along with their knowledge and experience as residents within the local area. In line with these priorities and our aim to explore participants’ thoughts about how to improve connections at a local level without a specific focus on technology, we designed a range of playful ideation (idea-generating) activities to scaffold workshop discussions. Choosing activities to maintain a ‘playful mindset’ was a central ambition in our design, as this has been identified as a key enabler when ideating [39].

Participants worked in small groups, with each group asked to choose one theme to focus on throughout the activities. We gave groups the option of completing one, some, or all of the activities, depending on which appealed to them and how much time they spent on each activity. All groups tried at least two of the three activities:

#### 3.2.1. Reverse Brainstorming

**Figure 1:**
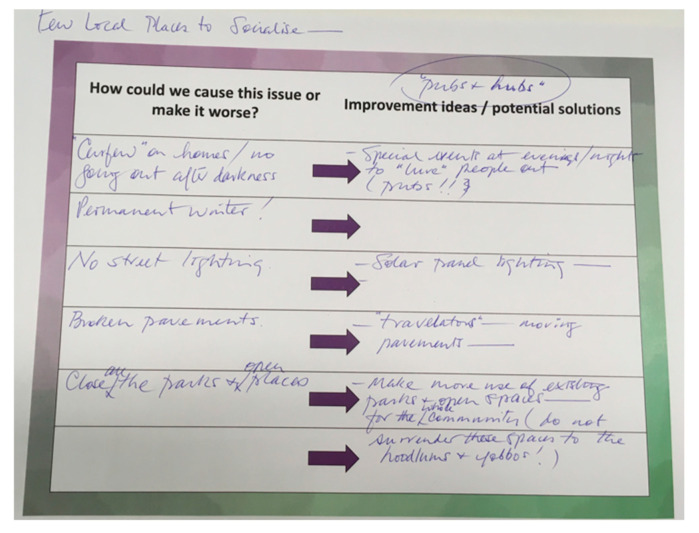
Example reverse brainstorming activity responses.

Participants were asked to generate ideas about how to cause the issue/theme or how to make it worse. This generated a list of problems or criticisms that participants were then asked to reverse or convert into positive ideas or solutions (Figure 1). An example idea from participants was to remove the internet. They then converted this into an idea to provide free internet access alongside TV licences.

#### 3.2.2. Character Activity

This activity involved imagining how a famous person or character (fictional or real) with a wealth of skills, resources or power might respond to the issue. One group chose Vladimir Putin, President of Russia, as their inspiration, with ideas that reflected their views on his leadership style, including mandatory socialising (e.g., meeting for a chat over a cup of tea or coffee) at particular times of day with street marshals to monitor and guarantee people’s involvement.

#### 3.2.3. Group Passing

**Figure 2:**
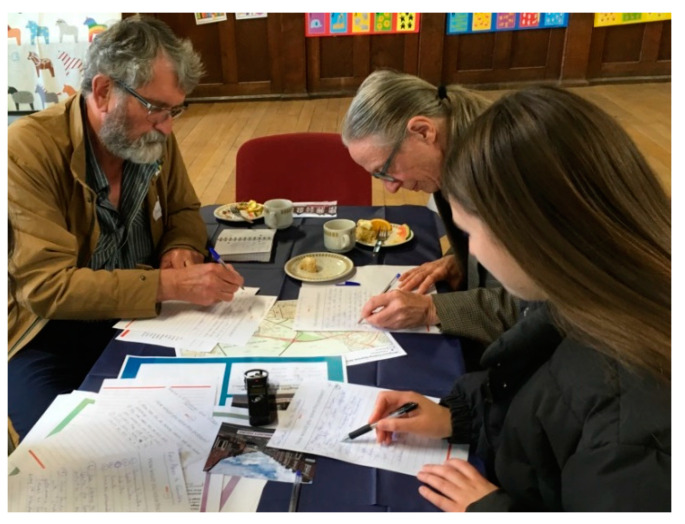
Participants developing ideas in the ‘group passing’ activity.

The third activity began with each group member writing an initial idea on a piece of paper which was then passed around the group for others to contribute to, comment on, or develop the initial idea (Figure 2). An example of this process was an initial idea to have more benches and ice cream vans driving round parks to encourage families with children to stay and chat. This resulted in the suggestion that the vans could double-up to provide other services like newspapers or bread, which might attract a wider range of people.

Data collection in the workshop comprised ideas written by participants on the templates provided (see Figure 1 for example data). All data were stored in a locked filing cabinet within an access controlled workspace. The workshop activities generated an extensive list of ideas and suggestions for facilitating social interaction within the immediate local area. Each group wrote down every idea that resulted from the activities they completed. After the workshop, we combined these ideas into one longer list and grouped and organised them under three overarching themes and 12 sub-themes that captured the overall range, content and types of ideas [37]. Themes and sub-themes were developed by two researchers (JL, TS) and then discussed with all members of the research team.

## 4. Findings: Phase One Interviews

As described earlier, the interview data were coded to explore (a) opportunities to improve connections at a local level, i.e., factors that had the potential to impact negatively on people’s geographically proximate social relationships in terms of quality, quantity or satisfaction; and (b) participants’ engagement with technology in relation to their social lives generally. The following sections outline the main findings in relation to each of these topics.

### 4.1. Opportunities to Improve Connections at a Local Level

In our interviews with participants, we adopted a place-based approach to focus in on social lives at a geographically local level. It soon became apparent that there were many aspects of the locality that participants were content with, or did not wish to change. For example, some described strong friendships and connections with local friends and neighbours that had endured over time. Others were actively involved in attending and/or organising local social events.

However, there were shared concerns and negative perceptions around local relationships, connections and characteristics of the area that offered opportunities for further exploration as topics to design around. Our analysis of the interview data specifically aimed to identify these opportunities to improve connections at a local level, by pinpointing factors that had the potential to impact negatively on people’s geographically proximate social relationships in terms of quality, quantity or satisfaction.

We report here on the four of these themes that were taken forward to the ideation workshop. These were chosen from a larger number identified, based on the criteria that they would be both appealing and generic enough for all participants to engage with, whatever their individual circumstances and experiences. Table 2 outlines the four themes, along with linked examples from the interview data.

Beginning with the first of the four themes, most participants reported that there were few places in the immediate local area that they could use for socialising beyond their own homes. They described how there was no central community centre in the area, and no clearly distinguishable main high street. Perceptions about the lack of local options contrasted with participants’ opinions about the venues, centres and cafés available in other areas where they felt that community spaces and cafés were prominent and actively used and adopted by people living there. Some participants were happy to socialise at home, but others saw this as too much of a burden or did not feel comfortable inviting people into their home. A noteworthy and unique characteristic of the local area highlighted by participants was the historic covenant on the land in the vicinity, preventing any licensed premises or pubs from operating. In the face of limited options in terms of usable spaces, local churches often hosted (or were booked to host) activities and events. However, this itself was a deterrent to some participants who felt uncomfortable attending events that had a religious connection—even if religion was not intended to be part of the event, such as a community café. Overall, the perspective was that the community’s physical features and built environment did not facilitate face-to-face social activities and interactions.

The second theme (not knowing neighbours well) did not apply to all interview participants. In fact, some participants described their neighbours as good friends. These interviewees lived in quieter, more spacious streets, accommodating larger houses with gardens. Other interview participants felt very disconnected from their neighbours. Those living in particularly ‘neighbourly’ streets were aware that their situations were unusual in the wider local area where different road and housing types and tenures were more dominant, and fewer longstanding residents were living alongside the same neighbours for extended time periods. Population churn, the movement of people in and out of streets, was perceived as a factor influencing the extent to which participants knew their neighbours. Growing families and the number of properties available to rent in the area were cited as reasons behind this movement. Streets were often busy with traffic—a factor that participants identified as not being conducive to unplanned meetings or chats with neighbours. While the physical proximity of neighbours potentially offered the most geographically close opportunities for social interaction, this had not translated into actual interactions for many participants. In particular, participants indicated that local issues of population mobility and transport routes contributed to the under-development of these relationships.

The essence of the third theme (a lack of shared community feeling) was expressed by many participants. Some attributed the absence of community to the area’s geographical characteristics and location within the wider city, including the proximity of a motorway and the absence of a central focal point, or main high street, in the area. Interview participants also commented on the lack of interaction between people of different ethnic and cultural backgrounds, despite the fact that the area was home to a diverse population. Some talked about how this had been a longstanding issue, first noticed when their children were at school. Together, both the physical environment and the population makeup of the area appeared to contribute to participants feeling that there were physical and cultural divisions within the geographical community.

The fourth theme illustrates the complexity of developing new connections and relationships that extend beyond acquaintanceship: activities on offer are not always conducive to socialising or making new friends. Even when participants were meeting people and seeking new friendships, these interactions did not often translate into deeper relationships. Some participants described attending regular or one-off activities where they felt that the type and format of sessions were not helpful for getting to know people. For example, the focus was on a particular activity so chatting was only possible during brief time periods while setting up or packing away. Another barrier was that some participants were more passive than others, and did not initiate conversations or connections themselves. In addition, participants mentioned that the same volunteers or people were often involved in several different groups and activities, resulting in a smaller pool of people to form friendships with. In other instances, it was simply that occasional casual conversations participants had with others did not result in deeper friendships or relationships that were sustained or developed beyond interactions at the events themselves, and individuals, therefore, remained acquaintances.

Taken together, these themes demonstrate clear barriers in, and characteristics of, local community connections. The themes capture issues that were impacting on the quality and quantity of participants’ relationships in the local area, offering opportunities for participatory design processes to address these.

### 4.2. Technology and Social Lives

Alongside identifying opportunities to improve connections at a local level, the other focus of our analysis of the interview data was on understanding more about participants’ existing engagements with technology in relation to their social lives. This engagement ranged from minimal (i.e., landline telephone only) to extensive (including social media, real-time audio/video interactions and applications).

We use eight central themes to capture participants’ accounts of the existing roles that technology played, or did not, play in their social lives. These themes, and examples of the data that support them, are outlined in Table 3.

The first theme about the role of technology in interviewees’ social lives focuses on its use to connect participants with people in geographically distant locations. In fact, many of the digitally mediated interactions described by participants bridged geographical distances. Applications and platforms such as FaceTime, Facebook and WhatsApp (along with traditional landline phone calls) were commonly used to keep in touch with friends and family located in geographically separate locations. Grandchildren were frequently mentioned as being a priority in seeking to connect face-to-face at a distance. While the financial savings of free long-distance technological connection were noted and appreciated by some, interviewees also reflected on the emotional value of being able to stay visually connected with loved ones. For Claire, this connection even changed her perception of the duration of time passing between in-person interactions, making it feel like she had seen her son in person more recently than was the case in reality. In contrast to those using technology to bridge distances in order to maintain existing relationships, Deborah was unusual among interviewees in that she had formed long-lasting friendships with people she met initially through the use of an online marketplace. As someone living alone in later life, she was using technology designed for one purpose (financial/accommodation transactions) to initiate and facilitate face-to-face interactions with strangers from geographically distant locations, offering the potential for developing new social relationships.

Our next theme encapsulates the role of technology in connecting family members and groups. Family relationships were frequently discussed as examples of connections that were supported by technology, through informal chatting, sharing photographs or stories and news about day-to-day life events. Family connections using technology ranged from group chats to individual messages, and instant short communications as well as ongoing asynchronous conversations. WhatsApp was often highlighted in this context, particularly for its usefulness in communicating with a group, and across generations. Examples included WhatsApp groups with interviewees, their children and partners, and grandchildren. These were sometimes longstanding groups for general communication, but at other times were set-up for a specific purpose, such as organising a birthday party. Cross-generational interactions were also perceived as improving the connectedness of family members who had previously felt ‘left out’ of family communications. John described the example of his sister, who was previously less connected with other members of the family but could now see photographs and hear about what other members of the family were doing, without them needing to make a special effort to include her. Technology was seen, in cases like this, as a solution to the barriers to instantaneous communication with family members with diverse and busy lives and routines. However, telephone calls were also important to participants as a way of keeping in touch, particularly with others who were nearer in age such as siblings or friends. In addition, Paul expressed his unease at the invasive nature of commonly used apps and platforms which, for example, access lists of contacts from the device they are using or collect data to support targeted advertising. His use of WhatsApp was ‘reluctant’ on this basis, but he acknowledged its usefulness in keeping in touch when his son was abroad, highlighting the trade-off he had to negotiate between privacy and connection.

We did not ask participants explicitly about the ways in which they chose to record social interactions or events, but the use of in-built cameras in mobile phones featured in participants’ accounts of the role of technology in their social lives. We have described this theme as ‘capturing and sharing images’. The ease of taking photographs with a smartphone in comparison to using a camera was noted by some participants, facilitating them in documenting social occasions. Moreover, despite his privacy concerns about the invasiveness of technology more generally, Paul valued the fact that he was able to recover digital images from an automatic cloud backup after he accidentally deleted photos (documenting an international trip) from his mobile phone. Photographs as mementos of experiences in participants’ social lives, like Paul’s trip, were treasured. Additionally, the act of sharing and receiving images was a central feature of participants’ digital interactions, connecting participants with events and experiences when they were not physically present.

After initially dismissing much technology (apart from FaceTime) as insignificant in her social life, Claire later reflected that it did play a large role in how she organised and arranged social events and interactions. The theme of ‘sharing information and making arrangements’ draws on these organisational uses of technology described by interviewees. Information was generally not necessarily shared on social networking sites or more visible platforms, but interactions commonly took place through instant messaging and other technological channels rather than solely in person. In fact, for Marie, there were additional benefits to using technology as a tool for organising or making arrangements with people. She preferred the control that it gave her in contrast with the unpredictability and social awkwardness she experienced when talking on the phone.

Technology was mainly described by interviewees in terms of its role as a tool for connecting, or supporting connections between, people. Conversely, several participants noted the ways in which technology itself was a dimension of their social life, offering an alternative to interactions with people. Perhaps because of its dominant focus on portraying human lives and activities, Jane felt that television was a more ‘personal’ type of technology. Patricia and Brenda watched television at times when other company or interaction was inaccessible. For Patricia, this was at ‘silly hours’ of the day or night, whereas Brenda described how she might watch television, DVDs or listen to CDs when she found herself alone or ‘down’. There were particular times when others living in her housing development were more likely to be spending time with family, such as weekends, where she used music or television as a strategy to deal with loneliness. At the other end of the spectrum, Simon tended to avoid face-to-face social activities and events with other people, preferring to spend time playing games or reading on his computer.

There were two main ways that participants described technology as contributing to disconnection in terms of social interactions and events: its prevalence as a platform for information about events; and its disruptive potential during face-to-face interactions. Sally used the internet but chose not to engage with social media for privacy and security reasons, but felt that this was increasingly disadvantaging her when it came to finding out about local events. She reflected on her reliance on other people to keep her informed, and the difficulties of being separate from the dominant route of information sharing via social media. For Sally, information sharing was happening in a way that excluded her, meaning that she missed out on attending social activities and events that she would have chosen to go to otherwise. In contrast, Liz highlighted the capacity of technology to disrupt social interactions themselves. She described both a friend’s extensive use of a smartphone, and purely the presence of a phone (in use or not), as disrupting face-to-face interactions and impacting on their quality. Sally’s and Liz’s accounts indicated a reluctance to allow technology to become pervasive in everyday life, balanced against a recognition that there were places and circumstances where it could be beneficial.

Along with concerns about the potential for technology to disrupt relationships, the positive impacts of technology in participants’ social lives were also, in some cases, accompanied by additional unwanted work. Our penultimate theme, therefore, centres around experiences of technological interaction as an additional ‘chore’. Sally described being ‘bombarded’ by messages, and she and others found their perceived continual need to respond and interact electronically to be a burden. The perpetual nature of communicating using interactive technologies such as email, texts and instant messages was also unpopular with some interviewees because of the amount of time it consumed. Responding was not perceived as an optional activity. Even if emails contained welcome content, the task of checking, opening and reading them was viewed as a compulsory individual task and responsibility. Catherine likened this to the responsibility to open letters that came through the post, rather than a choice or pleasurable activity.

Our final theme sums up participants’ thoughts about not needing digital technologies. More traditional technologies such as the telephone or television were commonly accepted as integral to daily life. In fact, their deep-seated role in participants’ social lives meant that they were often no longer considered or mentioned (by participants) when talking about technology. Instead, participants tended to focus on newer digital technologies such as social media, applications and email. Regarding these more modern technologies, there was a sense for some participants that they were unnecessary. For example, when talking about social media, Liz explained that she did not ‘think there’s a gap that I need them.’ Christopher used the internet and email but did not consider it necessary to go online to find out about local social events as he was exposed to paper-based publicity, such as posters and flyers, as well as information via word-of-mouth. For Judith, the whole idea of using a computer or the internet was superfluous when she could instead rely on her family for support, asking them for anything she needed.

Overall, technological connections were predominantly bridging distances, with existing local technological connections less obvious. Technology was mainly seen as a tool to be used to make connecting easier where there were needs, barriers or ‘gaps’ (geographical or generational distances, difficulties sharing information, capturing images, avoiding uncomfortable face-to-face interactions), but not at the expense of disrupting desired face-to-face interactions or in situations where technology was seen as unnecessary (other strategies would suffice). In addition, the additional work required to use technology as an aid to connection was an unwanted consequence. Willingness to use technology depended on balancing the positive and negative aspects.

## 5. Findings: Phase Two Workshop

### Exploring Ideas to Increase Opportunities for Local Social Interactions

As described earlier, the workshop was designed to build on the findings from our interviews. An extensive list of ideas was generated through our ideation activities, which we combined and organised under themes and sub-themes. Table 4 summarises the themes and sub-themes identified in our analysis of the written workshop data. Participants commented that the workshop had been enjoyable and thought-provoking—an outcome that supports us in challenging ageist stereotypes of older people as unable or unwilling to engage in creative, disruptive or wild thinking.

The three main themes we use to understand the workshop data are: social spaces and places; processes to promote social interactions; mechanisms to drive change. These themes capture different dimensions of participants’ ideas for facilitating social interactions in the local area. Ideas varied in both scale and scope (see Table 4 for examples).

The first theme describes ideas that related to the physical environment and developing spaces and places to promote interactions. The proposed changes were either to directly provide locations for organised or informal activities to take place, or to change environmental factors to increase the likelihood of people meeting and connecting in their everyday lives. Ideas for developing locations for activities included making better use of existing spaces as well as creating new spaces or places. Residents suggested taking advantage of the large areas of green space that were nearby and using them in new ways. They also thought that new community premises, such as a community centre, would be helpful. Ideas to change other environmental factors included improving the environment for pedestrians and improving security of tenure to increase the length of time that people are resident in the same location before moving home. While some ideas residents suggested were more generic, others were particularly context-specific. Participants drew on their local knowledge to consider what resources in the local area could be used, and identified other resources that were lacking.

The second theme brings together ideas that participants had for processes and actions that could play a part in promoting social interactions. These included: prioritising engagement within the wider community to develop ideas; connecting different groups with each other; improving provision of information about events and activities in the local area; connecting people with locations and activities in the city centre; and focusing particularly on making use of proximity as a tool in the process of connection. Encouraging people to walk in the local area more often, and setting up hyper-local events such as street meetings, were examples of ideas to facilitate people in connecting with others living in close proximity. Participants’ ideas emphasise the importance and desire for strong relationships at a local level, particularly building on the existing work and connections of volunteers and groups that they were aware of.

The third theme considers what types of mechanism could be used to drive change and engagement by local people, in order that involvement in supporting social connections is seen as an attractive opportunity. Participants’ ideas included the use of cooperative initiatives to develop or run transport services or community spaces, and incentives for small businesses to make the local area an attractive place to set up or move to. They also suggested that incentive schemes for local residents (such as loyalty cards or credits) to participate in local activities would encourage people to maintain involvement. Participants proposed that making a public commitment to community work could not only increase the contributions made by individuals within the local area, but also contribute to an increased sense of community. Taken together, these ideas portray a community with actively engaged members working to make positive changes, that directly and indirectly lead to individual connections being strengthened.

We take forward one example sub-theme from each of these three main themes for further consideration in the second half of the discussion section of this paper, in order to begin thinking about how technology might contribute to supporting these types of initiative, as well as noting some of the challenges that would need to be addressed in designing such technologies.

## 6. Discussion

This paper makes a case for adjusting the design process to accommodate a bottom-up component that precedes design of technological outputs. We begin our discussion of the findings from this study by considering the interview data, and their position in relation to wider debates and literature around technology and social interaction in later life. We then move on to discuss what the ideas generated by workshop participants offer in terms of implications, scope and challenges for future technology design around social connectedness, particularly when considered in the context of the interview findings. We use three sub-themes from the workshop (making better use of existing geographical places and spaces; focusing on proximate relationships; community driven/commissioned or cooperative initiatives) as examples to avoid our discussion of implications and challenges for future technology design being too generic, and to ensure that our focus remains on designing in the particular context of our research community and participants.

Within an age-friendly context, our analysis of interview data identifies a number of opportunities to design for increased social connectedness within local communities. Participants felt that: there were few local places to socialise; they often did not know their neighbours well; there was an absence of shared community feeling; social activities on offer did not always lead to socialising or making new friends. In a policy and practice environment where technology-based initiatives are increasingly perceived as offering huge potential, our findings highlight the importance of age-friendly approaches that are grounded in the local context [1,2]. This has become even more apparent during the COVID-19 pandemic, which has exposed the need for digital connection as an alternative to face-to-face interactions. Similarly, finding new ways to connect, including with people in proximate locations, has become even more important in ways we did not anticipate when conducting this study. Every community is unique, so designing to optimise social connectedness at a local level requires understanding and recognition of context-specific characteristics. In addition, taking account of the social and structural particularities of places gives insight into meanings and functions that are the result of cumulative experiences over time [40]. In our study, the geographical layout of the community, restrictions on licensed premises and population churn were all factors that participants highlighted as playing a role in disconnection. However, these issues can also be seen as ‘leverage points’ where interventions could afford the greatest benefits within a specific local context [40].

Our interview data also contribute to understanding more about how older people use and perceive technology in their social lives. Unlike Dickinson and Hill’s findings in 2009 that older people did not engage with instant messaging or other forms of computer technology aside from email [29], participants connected using a range of methods and formations of communication. Family connections using technology ranged from group chats to individual messages, and instant short communications as well as ongoing asynchronous conversations. Participants were not necessarily using social networking sites to share information, as Righi et al. [30] also found, but in our study these information-sharing interactions were commonly taking place through instant messaging and other technological channels rather than solely in person. These findings reflect changing levels of digital connection for older people in the UK [28] and emphasise the need for HCI to reconsider longstanding stereotypes of older people as digitally inexperienced or uninterested [13]. The COVID-19 pandemic has provided further evidence to counter these outdated stereotypes, with many older people embracing technology to facilitate connections with friends and family at a time when face-to-face meetings have been restricted.

Yet, while participants in our study made regular use of technology to support their connections with others, this use was carefully considered. Technology was not, in itself, an attractive prospect unless it was perceived to fill a ‘gap’ and the ‘chore’ of using it did not overly impact on everyday life. Similarly, Lindley et al. reported that older people were cautious of the time commitments required to use technologies, although they also used technology as a way to manage levels of contact and control their own availability to other people [25]. In addition, participants in our study were aware of the potential for technology to contribute to disconnection. Waycott et al. [41] reflect that the mismatching of values and assumptions guiding a technology-based social intervention with those of the older adults participating in the evaluation, noticeably contributed to individual decisions not to participate. In an increasingly digital society, our findings again indicate the importance of design processes that are in tune with the perceptions and values of older adults.

Marston and van Hoof draw our attention to the fact that the World Health Organization’s age-friendly cities model does not explicitly consider the role of technology [1,42]. By adopting a lens of age-friendliness, studies like ours can ensure that methods and processes are rooted in opportunities, concerns and ‘gaps’ that are relevant and engaging to participants. Consequently, we put forward an amended definition that highlights the need for explicit and thoughtful consideration of the role of technology in an age-friendly setting:

Underpinned by a commitment to respect and social inclusion, an age-friendly community is engaged in a strategic and ongoing process to facilitate active ageing by optimising the community’s physical, social and digital environments and its supporting infrastructure.

Another contribution of our work comes from its findings about the potential for technology to contribute to building and strengthening connections in geographically-bounded communities. The combination of shared local concerns and opportunities for improving connections, combined with the knowledge that technology was infrequently used to sustain or support local connections, suggests this is a design space worth exploring. Participants in this study were comfortable using digital technology to stay in touch with friends and family in geographically distant locations, particularly to maintain close family connections. Kharicha et al. also found that engagement with the outside world by landline telephones and computers was an important strategy adopted by older people experiencing loneliness [43]. For this reason, it would seem plausible that technology to facilitate local, proximate, connections and social lives would also be acceptable, should it fill perceived gaps and not lead to unacceptable levels of additional effort.

The methods we used in the workshop were intended to encourage ‘playful’ creativity, and they were successful in their purpose of generating a wide range of ideas as well as being acceptable and enjoyable for participants. In future, we would consider adapting these methods to reduce their paper-based nature, further enhancing their potential for prompting creative thinking by participants. Exploring options beyond face-to-face participation may also be important in the context of COVID-19 and its aftermath.

Drawing the interview findings together with one sub-theme from each of the themes we used to organise the ideas generated by workshop participants, we suggest a number of ways in which technology might support greater face-to-face connection in local community contexts and operationalise local people’s ideas. By deliberately not placing technology in the foreground in the workshop, we contend that participants’ ideas (technological or otherwise) about how to tackle local issues are more likely to align with their own values and perceptions, meaning that any technological needs that arise from these suggestions will be filling ‘gaps’ rather than technology being introduced as the automatic interface in connection. We maintain that design processes and spaces should be context-specific and bottom-up, but summarise general implications that offer scope for further exploration and consideration in community settings.

### 6.1. Making Better Use of Existing Geographical Places and Spaces

Workshop participants expressed interest in re-purposing spaces in the local area that they felt were underused, or offered potential as social spaces. This ranged from using existing green spaces or buildings on a permanent or temporary basis, to creating new spaces and places for social activities and events. A real-life example of creative use of space by older people that challenges expectations and norms was the transformation (for one night only) of a nightclub in Manchester, UK, into a night-time venue reserved for older people [44]. In our study, there were suggestions that spaces could be acquired or managed by groups of local residents as cooperative initiatives. Such work is ongoing in virtual spaces by older people in the UK creating a radio network [45]. Other adaptations to the built environment were also suggested by participants to improve suitability for pedestrians. However, operationalising these ideas and coordinating the input of the local community presents challenges at many levels. While online platforms to facilitate community commissioning of digital services exist [46], it is not immediately clear that these tools and processes would translate to local community commissioning of resources and events. Moreover, it is unrealistic to expect the required intense interaction with such digital platforms, leading to the need for alternative situated means of participating and engaging in the processes. Given the interest by study participants in leveraging local infrastructures and spaces, it is plausible to consider situated artefacts that would mediate between local, physical, and online engagements. For example, PosterVote is an innovative electronic polling system aiming to provide easy electronic voting for communities [47]. A traditional poster is augmented with buttons that can be pressed by community members to register digital responses to questions on the poster. Providing infrastructure for residents to have greater input and control over the provision of their immediate local environments would facilitate their participation in the process of age-friendliness at a community level.

### 6.2. Focusing on Proximate Connections

While our workshop focussed on connections at a local i.e., electoral ward level, some discussions were about connecting with people who were located very close nearby or even physically ‘connected’ by living on the same street. In fact, two participants expressed surprise on discovering that they had both been living in the area for many years a few houses apart on adjacent streets, yet they had never interacted before. Concerns about safety, privacy and possible lack of interest by others were mentioned as barriers to interventions at a street level. In recent years, we have witnessed a surge in location-based and serendipitous dating/meet-up services and networks (i.e., Tinder [48]). The core functionalities of these technologies are the abilities to discover similar individuals in your local area; privately extend an invitation to initiate a conversation; whilst maintaining a degree of privacy and safety through the network’s services (not revealing personal details such as address or phone number). Such solutions would have scope to support the hyper-local match-making of friendships within communities. However, our research showed that participants were not using existing online services designed to develop new relationships, indicating that these did not appeal. This is echoed by findings that older people who were lonely did not report using the internet to cultivate new friendships, despite using telephones and computers to engage with the outside world [43]. In fact, one participant, Deborah, had instead capitalised on the ability of an accommodation matching platform to facilitate face-to-face interactions in her home with strangers, who then had the potential to become friends. The opportunity for such encounters (through mutually beneficial financial, or other resource, transactions) to result in long-lasting friendships is an area for further exploration. In particular, it would be interesting to consider how these types of interaction could be translated into a purely local context, given that Deborah’s formation of new friendships contrasts with experiences of those in our study who attended regular local activities but did not find them conducive to making friends.

### 6.3. Community Driven/Commissioned or Cooperative Initiatives

The findings from our study indicate an opportunity for design around community or cooperative ways of addressing local transport gaps. A number of ideas generated by workshop participants related to improving transport in the immediate local area in order to facilitate connection to physical spaces and locations to meet other people. Community or cooperative initiatives were suggested as one option, or mechanism, for driving new models of transport in the area. Volunteer-run minibus and car transport did exist in the local area, but these prioritised ‘essential’ travel such as hospital appointments and did not have the flexibility that participants thought important. While existing schemes (e.g., StreetBank [49]—a website that facilitates possession sharing and borrowing between neighbours) have been successful in meeting other needs at a very local level, hyper-local journeys in suburban communities outside busy city centres are unlikely to offer sufficient cost/profit ratios to be attractive to existing sharing economy or peer-to-peer services such as Uber. A small number of demand responsive transport (DRT) schemes are running in the UK, and in theory sound promising. However, it is notable that a DRT service actually operated in our study area in the past, but closed in 2011 [50,51]. Similarly, existing bicycle sharing schemes rely on scale of use within large communities or cities to remain profitable, but in contrast, restricted access to a smaller population might reduce the risk of damage and loss experienced by larger scale operations. Consideration of what a hyper-local transport system might look like would include questions about who might provide and use the service, and what their incentives would be. Participants in this study also suggested teaching, learning and training opportunities as potentially playing a role. This is another avenue for exploration in future technology design which could serve the dual purposes of creating new connections between those learning and teaching, as well as the transport itself facilitating connections between people living in the area.

## 7. Conclusions

Our study adopted an age-friendly, bottom-up approach to explore opportunities for facilitating social connectedness for older adults in a local community context. We focused on specific community issues that could be addressed and considered the physical, social and structural mechanisms (potentially mediated or supported by technology) that might offer routes to tackling these. By understanding more about our participants’ current use and perspectives on the role of technology in their social lives, we highlight a need for design work to reduce emphasis on technology as the interface between people. In contrast to previous work, we focus on connection between people in geographically close locations. We also demonstrate the importance of understanding the specific local context within which any technological interventions will take place. Our findings reflect changing patterns of technology use among older adults in the UK, suggesting that adoption of new technology is acceptable when it fills gaps and does not create intrusive levels of additional work or contribute to disconnection. Our modified definition of age-friendliness highlights a need for the explicit and thoughtful consideration of the role of technology. We identify topics for consideration by those seeking to design with local communities, and make the case for an age-friendly approach to designing (digital) interventions to address social connectedness in later life.

## Figures and Tables

**Figure 3:**
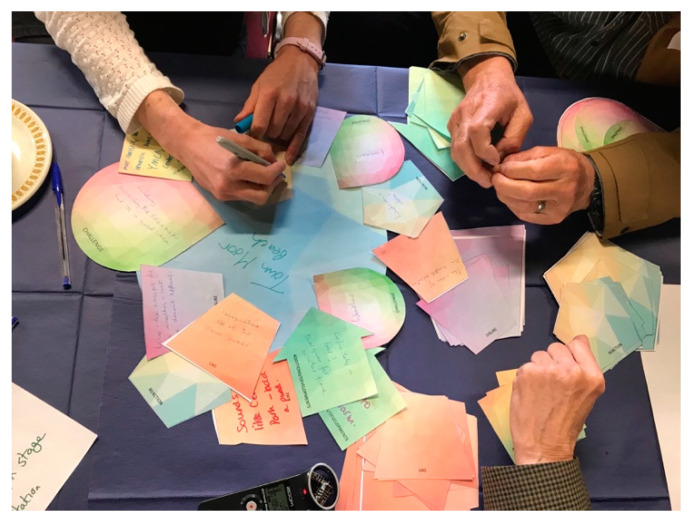
Participants generating an idea to develop a beach and water feature on an existing outdoor area of green space.

**Table 1:** Sociodemographic characteristics of sample.

	Number of Participants *n* = 22
**Age group (years)**	
60–64	1
65–69	5
70–74	7
75–79	4
80–84	5
**Sex**	
Female	15
Male	7
**Ethnicity/Nationality**	
White British	21
Asian British	1
**Living Arrangements**	
Living alone	8
Living with one other person	13
Living with more than one other person	1
**Marital Status**	
Married/Long-term Partner	14
Single	2
Divorced/Separated	1
Widowed	5
**Current Work Status**	
Retired	21
Part-time Work	1
**Recruitment Source**	
Community café	14
Electoral ward annual public meeting	2
Local church	1
Local organisation run by, and for, older people	1
Local wellbeing charity	1
Sports club	1
Referred by another interviewee	1
Gardening association	1

**Table 2:** Themes relating to concerns and negative perceptions about local relationships, connections and characteristics of the area.

Theme	Example Data
Few Local Places to Socialise	*“One of the things that’s wrong with [this area], I would say, is that there aren’t many ‘getting out of your house and meeting people’ places about.”* (Claire)
	*“I think it’s a shame that there isn’t a hub, a community hub type thing, because I witness what seems to be the backend of beyond […], where they have very, very active women’s institutes and community centres. [Here, there’s] a very small parade of shops and there’s not the communal coffee shop type place like there is in [another local electoral ward in the city].”* (Liz)
	*“I often think it would be quite nice to live somewhere where if you wanted to in an evening, […] just once a week, just a local pub you could pop into and meet people but of course that goes back to the covenant […] that there can’t be any public houses within a certain radius.”* (Catherine)
	*“There are not an awful lot of facilities here. If we were religious, then yes, there are churches here, but we’re not, either of us. […] I wouldn’t be interested if it was a church thing, no, no.”* (Alan)
Not Knowing Neighbours Well	*“I’ve lived in this street for 48 years. So it’s a street where there is a lot of movement. A lot of young couples come, they’re working couples, with no family and they’ll come for a couple of years. Then, there will be a baby come along and then they want a house with a garden. So they’re always on the move.”* (Sheila)
	*“I used to know people who lived in the next to end house. And I know the woman opposite, but I’m not particularly friendly with her. The busyness of the road doesn’t make it that conducive to… You know like you can’t really have a street party on this street. So I wouldn’t say I know a lot of people well around here.”* (Deborah)
Absence of a Shared Community Feeling	*“You can’t talk about the [local area] community, you just can’t, there isn’t one. There are people living in [the local area].”* (Alan)
	*“I think in some ways this [area] between the east central motorway and even going up towards [one of the main roads], it kind of hasn’t got a soul to it, it doesn’t have a kind of focal point, so it’s a bit of an amorphous area. I think an area that has a strong focal point seems to do better.”* (Paul)
	*“The degree of integration is not great. […] It is a source of concern that there is so little contact between the immigrant population and other folk.”* (Christopher)
Activities on Offer not always Conducive to Socialising or Making New Friends	*“There’s no friends, just had one lady who teaches me […] I forget her name. (Laughter) I’ve never contacted anybody from my class.”* (Rizwana)
	*“I’ve been attending a yoga class […] for years and years and years. […] But there is never an opportunity to have a chat to people. You might just be able to say, ‘Are you alright?’ as we’re packing up, you know, ‘I haven’t seen you for ages’, but there’s not an opportunity to chat with people.”* (Sally)

**Table 3:** Themes relating to the current roles of technology in participants’ social lives.

Theme	Example Data
Connecting across Geographically Distant Locations	*“We do FaceTime our son a lot. And actually, a friend in Japan and these friends in Portugal. It’s the easiest way. […] Well, it is nice to see people. […] Then we can show them that we’ve got the snow or whatever. I think it gives you more than talking on the phone, for sure. Yes. Especially looking at grandchildren and they [waving gesture], ‘Hello, Nana’.”* (Liz)
	*“But FaceTime, I love it. I really love that, because [Son] is in the States. And we must speak to him about four times a week actually. […] I love the fact that you can see him. You really feel that the person is there. I think, basically, I’d would be kind of heart broken, because he’s there, if it wasn’t for that. I find that hugely, very enjoyable and very good. When we go to see him, I kind of feel – while it’s lovely to see him in person – ‘It’s not been that long really’ because of…”* (Claire)
	*“I heard about Airbnb and thought, ‘I’ll try that and, you know, I could use it as a stop gap until I find somebody suitable to live here.’ Then I just liked it, […] it’s enjoyable, mainly nice people. I’ve made some friends. I’ve got invites to go… […] people who came as Airbnb guests and who have invited me to go and stay with them.”* (Deborah)
Connecting Family Members and Groups	*“Oh, WhatsApp all the time, with the family. We’ve got a family group. […] It would be a strange day when I didn’t get several WhatsApp messages from other members of the family, even if it’s only pictures of what they’re doing. ‘Here’s my dinner.’ […] I mean, I WhatsApp-ed them all last night when I’d been to the cinema, telling them what I’d seen and telling them to go and see it.”* (Christopher)
	*“I know at Christmas time myself and [son 1] and [daughter] had a group to discuss what we were going to do about [son 2]’s Christmas present. My eldest son has got a little girl, 18 months old, and when she was coming up to a year he had a family group to discuss her birthday party. […] So just anything where you want a group of you it’s just handy.”* (Catherine)
	*“We WhatsApp people all the time, we’re on a WhatsApp group as a family, we’ve got two, one for just my wife and I and the kids, and one for the wife and I, the kids and my sister. I think that’s great, because my sister’s becoming more isolated, she’s 82 with health problems, so she knows what’s going on, because she gets the WhatsApps, she gets the pictures. She always thought she was being left out until that, and instead of having to ring people round asking what’s happening or tell them about something, you just WhatsApp it.”* (John)
	*“They basically log your life and your habits […] for targeted advertising for people. […] So it can be quite invasive […] I reluctantly loaded the WhatsApp thing into my phone because of [son’s name] going to Singapore, it was handy when he was wanting to check up on one or two things back and forth. […] As soon as I downloaded it, it read all my contact list. […] So when I opened up the WhatsApp app I see all my contacts so it was a bit scary.”* (Paul)
	*“My sister, I talk to her, she lives down in the Midlands. I talk to her every now and then, every couple of weeks or so. Either she rings me, or I ring her.”* (Martin)
Capturing and Sharing Images	Marie: *“It’s got an excellent camera. I use it as a camera because I’m useless at taking photographs otherwise.”*
	Simon: *“See, if Marie uses a camera to take somebody’s photograph, and eventually either cuts them in half or chops their head off, you know, which is-But, with this phone, it’s absolutely brilliant.”*
	Marie: *“Yes, yes.”*
	Simon: *“The pictures that she’s taken when she’s been on holiday and things, absolutely superb.”*
	*“I get loads of photographs of the children when they’re opening birthday presents. Their mother takes a photograph and sends it with a comment on what they said when they were trying on things.”* (Lynne)
Sharing Information and Making Arrangements	*“Actually, it is more important than I’m making out because we’re always making arrangements. […] You have no idea how difficult it is making a weekend arrangement with people who are constantly out of the country. […] That’s another thing that’s important, I don’t know why I’m even saying it’s not important. We do go away quite often, so I do use technology to organise where I’m going and what we’re doing and what you can do while you’re there, basically. So yes, I do use technology quite a lot at that. It’s probably very important.”* (Claire)
	*“Because, I’m not very good. If I have to make a phone call to somebody, I’m terrible. If I’ve got to make, like, a cold call to somebody, ‘Can you phone so and so to ask them to do this?’ I’m going, ‘Do I have to? Do I have to?’ […] and I hate it. I’ve always hated the telephone for making phone calls out. Texting is a lifesaver. You can text whenever you want, when you feel like it, and you’re not having to speak to somebody and say the wrong thing. So, that’s what I like about that technology.”* (Marie)
Offering an Alternative to Interactions with People	*“television is something […] that is more personal than other electronic things.”* (Jane)
	*“Well, probably a Sunday, unless on the odd occasion [son] is here, because there aren’t any social things going on and most of the people here [in this housing development] probably have their children around them or their grandchildren, which I haven’t got, plus the fact that sometimes just your general health, if you just feel down generally, that makes you feel lonely as well. […] I put on music sometimes because I find it uplifting […] or a Pam Ayres DVD, which is funny, something that just makes you laugh or the music uplifts you.”* (Brenda)
Contributing to Disconnection	*“My friend had been [to a local activist/campaigning event] on the Friday, and because I don’t use social media I just hadn’t heard […] It did make me think, ‘What would I have to do not to miss the way information about things like that is circulated?’ […] To find out information about what’s on, you can’t really even Google, because if it’s on social media it doesn’t come up as a website. […] So there is the issue of becoming more isolated because the information sharing is happening in a way that I’m not part of.”* (Sally)
	*“[Our friend] has two phones and she’s always on them. I find that quite difficult. When she’s eating here, she’s not allowed phones at the table. She obviously finds that really difficult because she lives her life on Twitter or whatever. And I just, I’m not criticising her. I just recognise it’s a different way to be. But when I’ve read recently about if you’ve got a phone near you, you behave differently from if you hadn’t. Even if it’s not ringing. […] If you carry it everywhere with you, you do actually behave differently. Your subconscious is aware that you might be interrupted. I can really recognise that, so it’s very interesting.”* (Liz)
Creating another ‘Chore’	*“I’m not that keen on technology. […] Whereas I’ve used it in the past for emailing friends, as a way of keeping in touch, I hardly have time… no that’s not… […] there are so many things that need attending to. You are just bombarded by people […] and you’ve got to read it, or respond to it, or whatever. And […] I don’t have that long. I’ve got my tea cooking or I’m just doing a quick check. I am not sitting down to be chained to a desk for the next five hours […] It does feel like a chore.”* (Sally)
	*“In my social life? I suppose emails, texting… I’m going to be really honest with you. I find getting involved… These friends […] they’re on WhatsApp and constantly… I made a conscious decision, “I’m not joining it.” I just can’t be doing with constantly… […] So yes, emails and texts, yes, heavily into despite myself because it seriously takes too much time. […] It’s the interactive ones that are… You just think, ‘People text you for nothing, don’t they?’ You think, ‘mmm…’ So, yes I use it, but sometimes unwillingly.”* (Claire)
	*“I keep saying to [husband], ‘Emails are really like your post,’ you don’t get so many letters through the post now, they come* via *email, so you do have to look at them.”* (Catherine)
Not Filling a ‘Gap’	*“We have no need to use them. Interesting, locally, take, for example, the concerts arranged now by the church. […] The church warden puts up a big poster on the railings, ‘concert this week’ […] They have noticeboards, and she puts stuff up there, we put up notices inside the church when the café is on, on a Thursday […] and we put flyers through each other’s doors. Organising local things here is by word-of-mouth and that, sort of, contact.”* (Christopher)
	*“I do not have a computer. I do not have a laptop. The family have always said, ‘You should get one.’ I said, ‘No. If I need anything…’ […] When I need something, I get it from my son […] and his wife, and my daughter and the grandchildren, you know, and they can all do it. So I said, ‘What is the point of me having one?’ […] So, no, I have no regrets at not doing it.”* (Judith)

**Table 4:** Themes from workshop data analysis.

Theme	Sub-themes	Example Data
Social Spaces and Places	*Making better use of existing geographical features and spaces* for social purposes, such as large areas of green space (e.g., Figure 3)	Longer opening hours e.g., library
		Marquees/undercover spaces in parks etc., for rainy days
		Make better use of open/green spaces for community activities e.g., exercise equipment, open a beach, more benches, ice cream vans to encourage use of parks
	*Creating new spaces and places* for social activities and events—organised and informal	New premises—big sports hall, comprehensive village/community centre
	*Adapting the built environment* to support pedestrians and encourage other non-car travel	Pedestrianise more areas/reduce speed limits to 20 mph, improve pavements, introduce more crossings and travellators/moving pavements
	*Considering housing issues* from a social and community perspective, including security of tenure and student accommodation	Social/community-focused strategy for housing e.g., reduce proportion of properties not for permanent residence, improve security of tenure/rent control, expand student halls of residence into area
Processes to Promote Social Interactions	*Improving communication strategies and publicity* within the local area	Improving communication strategy/publicity e.g., television appearances, community newspaper/flyers (with rotas for delivery), local council to focus on one ward in turn in Council magazine
	*Making links across ethnic social groups*	Link between ethnic groups to reduce divides along religious/ethnic lines in community
	*Engaging with the wider community* to share ideas and seek feedback	Wider community preference-seeking around how to solve issue of few places to socialise e.g., questionnaire and workshops
		Maximise work of existing social groups
		Monthly meetings for volunteers to pool ideas
	*New transport options* to support travel in the immediate local area and into the city centre	Frequent, small scale local transport e.g., minibus every 10 min
		Extend the metro into the area to improve access to city
	*Focusing on proximate relationships* i.e., at a street level or between those volunteering at the same events, as well as at the community level	Encourage greater walking in area e.g., parents taking children to school
		Encourage volunteers to build friendships/relationships outside volunteering activities/context
		Street level interventions e.g., street meetings/cups of tea, annual events
Mechanisms to Drive Change	*Community-driven/commissioned or cooperative initiatives* around social spaces, information provision, transport and learning/training	Community/cooperative/volunteer-run hospitality venues
		Buy a property on a co-operative basis and use as community resource/café/party venue
		Community uber-style, tandems/sidecars or other forms of ‘fun’ transport, bike sharing, motorcycle lessons—teaching/learning/using transport
		Cafes that also operate as training kitchen for cooking healthily, training in basic work skills by involvement in running community hub
	*Incentives* to: sustain and attract small catering and hospitality businesses to the local area; encourage local people to participate in social activities	Increase incentives for small catering/hospitality businesses e.g., no rates/taxes for first years after opening
		Happy hours in cafes etc., with free tea/coffee/cake, sponsored by local businesses
		Credits for free attendance at social activities for residents e.g., swimming pool on particular days/times/a month, extra credits could be earned through volunteering
		Dedicated time slots for social and/or physical activity/exercise time
	*Finding ways of improving the commitment and contributions of individuals to the local area* to create and sustain a sense of community	Commitment of individuals to community e.g., minimum number of community work hours/community service and strategy to deal with those who do not contribute, volunteers to supervise weekend sporting activities for children, create sense of community between residents/students
